# Manufacturing of Sustainable Composite Materials: The Challenge of Flax Fiber and Polypropylene

**DOI:** 10.3390/ma17194768

**Published:** 2024-09-28

**Authors:** Gianluca Parodo, Luca Sorrentino, Sandro Turchetta, Giuseppe Moffa

**Affiliations:** Department of Civil and Mechanical Engineering, University of Cassino and Southern Lazio, 03043 Cassino, Italy; sorrentino@unicas.it (L.S.); turchetta@unicas.it (S.T.); giuseppe.moffa@unicas.it (G.M.)

**Keywords:** flax fibers, polypropylene, sustainable composites, hot press molding

## Abstract

The widespread use of synthetic composite materials has raised environmental concerns due to their non-biodegradability and energy-intensive production. This paper explores the potential of natural composites, specifically flax–polypropylene, as a sustainable alternative to traditional composites for semi-structural applications. In fact, the mechanical properties of flax–polypropylene composites are similar to synthetic ones (such as those made with E-glass fibers). However, processing challenges related to fiber–matrix interaction and material degradation necessitate suited process parameters for this sustainable type of material. For this reason, this review highlights the importance of optimizing existing manufacturing processes, such as hot press molding, to better accommodate the specific characteristics of polypropylene–flax composites. By refining the parameters and techniques involved in hot press molding, researchers should overcome current limitations and fully capitalize on its potential to produce composite materials of optimal quality. Therefore, a comprehensive literature assessment was conducted to analyze the properties and processing challenges of flax–polypropylene composites. Key process parameters affecting the material’s performance are identified and discussed. By optimizing process parameters for flax–polypropylene composites, it is possible to develop a sustainable and high-performance material with a reduced environmental footprint. Further research is needed to scale up production and explore different applications for this sustainable composite material.

## 1. Introduction

Flax-reinforced polypropylene (flax–PP) composites have emerged as a promising material in secondary load-bearing applications, particularly within the automotive industry, due to their high toughness and low density [1,2,3,4,5]. This trend towards bio-composite materials is further driven by regulatory pressures that favor sustainability and recyclability [6]. These materials present a potential alternative to glass fiber composites [3,7,8,9], offering the advantages of a lightweight material (40 to 60% less dense than glass fibers [10]) and environmental benefits. As a natural, renewable resource that is widely available globally, flax offers considerable potential for the development of biodegradable and recyclable composite materials. Such materials may potentially supplant non-renewable fossil fuel-based polymers in a variety of applications, thereby contributing to the development of a more sustainable and circular economy [11,12]. Additionally, flax crops have the capacity to sequester carbon dioxide during growth, thereby further enhancing their environmental benefits [12].

In comparison to glass fiber–PP composites, flax–PP composites demonstrate superior specific load, energy absorption, and impact damage tolerance [13]. Furthermore, flax–PP composites have been demonstrated to outperform thermoset-based composites, such as those with epoxy or polyester matrices, in terms of impact damage tolerance [14]. Polyester-based flax composites, however, demonstrate higher failure strains and fiber pull-out mechanisms, leading to improved impact resistance [14].

The recyclability of thermoplastics, unlike thermosets, makes them a favored choice for sustainable material production, as they can be reprocessed multiple times [15]. Among the most widely used and recycled thermoplastics, polyethylene and polypropylene are particularly suitable for vegetal fiber composite manufacturing owing to their low processing temperatures [16].

Flax-reinforced polypropylene composites offer a number of advantages over other natural fiber-reinforced plastics. In comparison to alternative natural fibers, flax fibers exhibit notable advantages in terms of weight reduction, tensile strength, and economic viability [17]. 

Polypropylene’s hydrophobic nature renders it an especially advantageous matrix for flax fibers, offering superior moisture resistance in comparison to hydrophilic thermoplastics such as polyamide [18]. This reduced susceptibility to swelling and degradation in humid environments is crucial for ensuring the long-term performance of natural fiber composites. While thermosets may initially demonstrate effective moisture resistance, their non-recyclability and environmental impact make them less appealing as options. Additionally, polypropylene’s lower melting point facilitates easier processing with flax fibers, further reinforcing its suitability as a preferred matrix.

However, the development of efficient processing technologies for flax–PP composites is impeded by the high viscosity of thermoplastic matrices, which hinders the rapid impregnation of fiber reinforcements. This challenge is particularly significant in the automotive industry, where manufacturing cycle times are constrained to a few minutes [19].

One strategy for addressing the issue of high viscosity is to increase the temperature of the resin. However, natural fibers such as flax are susceptible to degradation at elevated temperatures, with a thermal decomposition around 200 °C setting a practical upper limit for processing [20]. Indeed, while natural fiber composites are often touted as recyclable materials, the recyclability of flax fiber composites remains a subject of considerable debate due to the susceptibility of these materials to degradation during both processing and recycling. This issue is particularly pronounced for flax fiber composites in comparison to glass fiber materials [21]. Furthermore, it is possible that the degradation of natural fibers may occur before the resin melting point, when the processing temperature is excessively high [22]. This is especially critical for flax fibers and polypropylene resin, as their respective degradation and melting points are closely aligned. Consequently, identifying the optimal processing parameters for PP–flax composites is a pivotal aspect of the manufacturing process. In particular, the processing parameters, including temperature, dwell time, and pressure, are decisive in determining the properties of plant fiber-reinforced thermoplastic composites. Consequently, ongoing research is necessary to optimize their performance [23].

In addition to the difficulties associated with high viscosity, flax–PP composites, like other WPCs (wood–plastic composites), encounter other obstacles, including poor dimensional stability and high hydrophilicity [24,25] which restrict their outdoor applications [26,27], as well as low fiber–matrix adhesion, which can impair their mechanical performance [2]. Enhancing fiber–matrix adhesion through the implementation of diverse chemical treatments has shown improvements in the mechanical characteristics of these composites, thereby rendering them an increasingly viable option for the aerospace and automotive industries [12]. However, debonding at the fiber–matrix interface persists as a considerable challenge, exerting a marked influence on the mechanical behavior of the composites [28,29]. Additionally, the mechanical properties of flax fiber reinforced composites are also influenced by fiber configuration (fabric, mat, yarn, roving, monofilament) and distribution [12], and moisture absorption, which can hinder the production of consistent and durable natural fiber composites.

The production of natural fiber–polymer composites has largely been based on manufacturing techniques that were originally developed for synthetic fiber-reinforced polymers. Methods such as resin transfer molding, vacuum infusion, and compression molding have been extensively utilized and have demonstrated efficacy in the fabrication of high-quality synthetic composites. However, the intrinsic differences between natural and synthetic fibers, including their chemical, mechanical, and thermal properties, present considerable challenges to the direct application of these methods. For instance, the hydrophilic nature of numerous natural fibers necessitates surface treatments to enhance compatibility with hydrophobic resins. 

Liquid composite molding (LCM) processes, particularly compression resin transfer molding (CRTM), have been recognized as cost-effective techniques for the production of composites, eliminating the necessity for semi-products. CRTM is particularly promising for producing automotive parts with short cycle times, thereby meeting the industry’s demand for efficiency. In a study conducted by Kim S. et al. [19], the fabrication of flax fiber textile-reinforced thermoplastic composites using the CRTM technique was investigated. The objective of the research was to optimize temperature settings and pressure patterns in order to achieve complete resin impregnation while minimizing fiber damage. The use of sequential pressure cycles, commencing with low pressure and subsequently increasing, was observed to be more effective than the application of constant pressure in ensuring uniform resin distribution. Higher temperatures promoted resin flow but also increased void formation. The application of elevated temperatures facilitated resin flow, yet concurrently augmented void formation. Approximately 215 °C was identified as the optimal temperature, which balances resin flow and fiber degradation [19]. While the CRTM method shows promise, its effectiveness is constrained by the uneven distribution of voids, which has a detrimental impact on the mechanical properties of the final product [19]. Indeed, the CRTM process is constrained in its ability to guarantee uniform resin distribution, particularly at the downstream end of the composite, where the fiber volume fraction is higher due to the compaction of the reinforcement during resin flow.

Alternative methods, including thermo-consolidation of semi-products (such as commingled yarns, powder-impregnated fabrics, and film stacking), have also been investigated. These methods entail the combination of solid thermoplastic polymer materials with fiber reinforcement in pre-mixed forms [30]. The utilization of semi-product materials such as film stacking or prepreg enables the implementation of a novel and promising consolidation technique, namely hot press consolidation [31]. This method is particularly well suited to reducing the energy consumption associated with composite material production, especially in comparison to traditional manufacturing processes [31]. While these approaches offer promising advantages, they are hindered by the requirement for pre-preparation of matrix-reinforcement materials, which can result in elevated manufacturing costs. 

This research aims to provide a comprehensive review of existing literature on flax–PP composite processing and to explore the potential of hot pressing as a more efficient and adaptable method for producing green components, especially in sectors that require high production rates.

## 2. Flax Fiber

### 2.1. Flax Fiber Structure

Flax represents a viable alternative to the conventional use of glass fiber reinforcements. Indeed, flax has a significantly lower density (1.40 g/cm^3^) compared to E-glass (2.56 g/cm^3^) [32], while maintaining comparable mechanical properties (Table 1). As a consequence of its lower density, flax composite materials exhibit a greater specific flexural stiffness than steel and aluminum, and their specific longitudinal tensile stiffness is superior to that of glass fibers [33]. 

In addition, flax is considered to pose lower health risks to workers in composite production. Unlike glass fibers, natural fibers do not induce skin irritation and are not suspected of causing lung cancer. This advantage is particularly significant given the ongoing debate surrounding the potential carcinogenicity of fine glass fibers [21]. While flax, as a natural fiber, can generate substantial dust, this issue is primarily associated with the initial stages of fiber extraction and is effectively managed in contemporary processing facilities [21]. 

**Figure 1:**
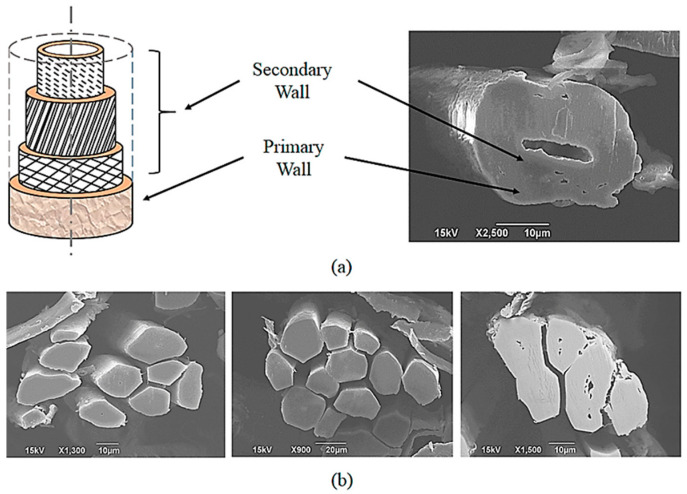
Longitudinal structure of a flax fiber cell (**a**); transverse cross-section of a flax fiber bundle (**b**) [35].

Flax fibers are derived from the Linum usitatissimum plant, a member of the Linaceae family. The stem contains bundles of elemental fibers. In practical applications, these bundles, known as technical fibers, are commonly utilized. Elemental fibers are characterized by a polygonal cross-section with rounded corners and multi-layered walls that surround a central hollow space, referred to as the lumen (see Figure 1). They are closed at both ends and typically measure 8 ÷ 51 µm in diameter (with an average of 19 µm) and 8 ÷ 69 mm in length (with an average of 32 mm). Technical fibers, which are composed of multiple elemental fibers, typically range in length from 0.3 to 1 m [34].

Generally, plant fibers are distinguished by an intricate cellular architecture. The fundamental structural unit, the microfibril, is a hollow cylinder comprising a primary cell wall, three secondary cell walls, and a central cavity [36]. Each of these layers contains cellulose, a semicrystalline polysaccharide that is the most rigid and strong organic component, significantly contributing to the fiber’s mechanical properties. Additionally, the matrix that embeds the cellulose is made up of hemicellulose and lignin, both of which play roles in the overall fiber strength [37,38,39]. Surrounding the bast fiber bundles is a protective layer that consists of pectins and waxes—substances that are not fibers themselves but serve crucial protective and adhesive functions. Pectins act as an adhesive between the cells, contributing to the cohesion and flexibility of the fibers, while waxes form a hydrophobic layer that protects against moisture and external agents [40]. At a more detailed level, the microfibril structure of cellulose exhibits alternating crystalline and amorphous zones, further influencing the mechanical characteristics of the fibers [39]. 

Hemicelluloses, which are branched and amorphous polysaccharides, are strongly bound to cellulose through the formation of hydrogen bonds. Despite having a shorter chain length in comparison to cellulose, their open structure, which contains hydroxyl and acetyl groups, results in partial water solubility and hygroscopicity, which consequently affect the fiber’s overall properties [38,39]. 

Lignin, an amorphous aromatic polymer, serves as a bonding agent within the polysaccharide network during cell wall formation, thereby enhancing the overall rigidity, durability, and strength of the cell wall [36,37,41]. Although lignin exhibits minimal water sorption, it provides protection and contributes to the overall mechanical stability of the fiber. Additionally, the waxy substances present in certain fibers, such as flax, can impact their wettability and adhesion characteristics.

Although the fundamental chemical composition of cellulose remains constant across plant fibers, variations in the length of the chains and the morphology of the cells differentiate the various fiber types. Furthermore, the structural and chemical properties of fibers are significantly influenced by factors such as plant maturity, environmental conditions, and processing methods. Given their intrinsic structure, plant fiber composites are particularly susceptible to high porosity levels, which significantly impacts their overall properties and performance. The issue is compounded by several factors, including the intrinsic hollow structure of plant fibers (lumen), the difficulty in bonding plant fibers with the matrix material due to their complex surface chemistry, the inconsistent shape and size of plant fibers, and the inefficient packing of plant fiber assemblies, which limits the maximum achievable fiber content [28].

Voids within natural fiber-based composites can be classified into several categories [19,28]. The first category is the lumen, which, as previously stated, constitutes the natural void within each individual flax fiber. In contrast to the other void types, the lumen is an inherent feature of the flax fiber structure, rather than a defect introduced during processing. Secondly, voids may also occur within the cortex, which is the outer layer of both individual flax fibers and fiber bundles. The cortex, which is primarily composed of pectin, hemicelluloses, and other binding substances [42], represents another source of fiber-inherent voids. The third category of voids, the fiber–matrix interfacial void, can be attributed to inadequate fiber–matrix adhesion or improper impregnation during the manufacturing process, as well as external mechanical stresses experienced by the composite during its service life [28].

The potential for blister formation on the composite surface represents a further critical consideration when assessing porosity. It is well established that water ingress into thermoplastic composites results in the degradation of fiber–matrix interfacial bonding, which in turn leads to long-term deterioration of the material’s properties. Suri C. et al. [43] investigated the phenomenon of blister formation in thermoplastic composites and its relationship with certain parameters, including material moisture content, exposure time, and thermal shock severity. The objective of the research was to elucidate the underlying mechanism of blister formation and its correlation with environmental conditions and material properties. The study yielded several significant findings. Firstly, it was established that water absorption represents a critical factor in the development of blisters. A direct correlation was identified between the extent of water uptake by the thermoplastic material and the severity of blistering following thermal shock. A critical water content threshold was identified, below which blister formation did not occur, irrespective of thermal shock conditions. Moreover, the research demonstrated that both the temperature and duration of thermal shock impacted blister formation. Similarly, minimum thresholds were identified for both parameters, beyond which blister formation was initiated. A linear relationship was observed between these parameters and the extent of blistering, which reached complete surface coverage in some instances. While the study offers valuable insights into the blistering phenomenon, it is essential to acknowledge its limitations. The characterization of the relationship between thermal shock parameters and blister evolution was based on a relatively limited data set. Moreover, although the study underscores the pivotal role of the weakened fiber–matrix interface resulting from water absorption in blister formation, further investigation is necessary to fully elucidate the influence of interface strength on this process.

It is of great importance to exercise meticulous control over the processing parameters in order to reduce the formation of voids during the manufacturing of components. This will result in a deterioration of the mechanical properties and an increased water uptake. By modifying these parameters, the permeability of thermoplastic resins through the reinforcement can be regulated, thereby reducing the overall void content.

### 2.2. Permeability of the Flax Reinforcement

The successful production of high-quality natural fiber-reinforced thermoplastic composites is contingent upon the effective impregnation of the fiber reinforcement with molten thermoplastic resin. The impregnation of a natural reinforcement is a complex topic, the intricacies of which are influenced by a multitude of factors, including the specific fiber type, the processing parameters employed, and the consolidation techniques utilized. It is challenging to ascertain permeability and impregnation times with precision due to the intricacies involved in accurately measuring the local fiber volume fraction and the necessity of elevated process temperatures to reduce polymer viscosity to manageable levels [44,45].

**Figure 2:**
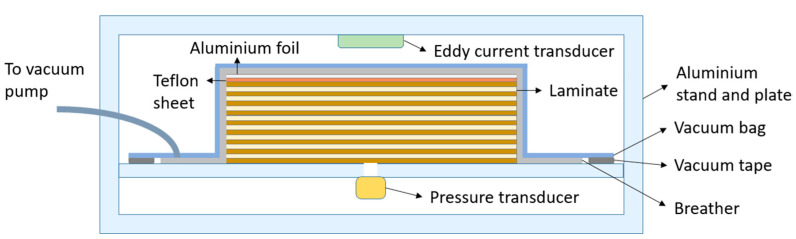
A schematic representation of the VAOC system.

In a comprehensive study, Kazmi et al. [31] examined the impact of diverse flax reinforcements and processing parameters on resin flow permeability during the consolidation of high-quality thermoplastic composites. The study’s findings offer insights into the mechanical properties and microstructure of these composites. The consolidation of the flax and polypropylene (PP) sheets was performed using the VAOC (vacuum-assisted oven consolidation) processing technique. Figure 2 provides a schematic representation of the aforementioned technique.

Three distinct flax reinforcements (fine twill, coarse twill, and bidirectional) were subjected to three different oven cycles (varying temperature and pressure profiles) to determine the optimal processing conditions:

- Cycle 1: The vacuum is applied before the heating phase and maintained at a constant level until the end of the forming process.
- Cycle 2: The vacuum is applied at the end of the heating phase and maintained at a constant level until the end of the forming process.
- Cycle 3: The vacuum is applied before the heating phase and maintained at a constant level until the end of the forming process. However, the holding time is reduced in comparison to Cycles 1 and 2 (the holding time for Cycles 1 and 2 is equal).

A key finding is that homogeneous fiber and resin distribution is crucial for preventing voids and achieving optimal mechanical performance. Microscopic examination of laminate cross-sections revealed that Cycle 1, which involved the application of vacuum prior to the heating phase, resulted in a reduction in resin-rich areas and an improvement in composite properties when compared to Cycle 2. However, the Cycle 2 laminates exhibited greater porosity due to the delayed application of the vacuum.

The cooling rate exerts a significant influence on both laminate thickness and fiber volume fraction (V_f_). The authors posit that a faster cooling rate results in thinner laminates with a higher fiber volume fraction (V_f_) due to reduced polymer crystallization. However, other studies have demonstrated that the cooling rate exerts an inverse effect on the matrix density [46,47]. While Cycle 3 reduced the manufacturing time for coarse and fine twill reinforcements without compromising the mechanical properties, bidirectional flax required the longer holding time of Cycle 1 for adequate resin impregnation. This suggests that the consolidation of bidirectional flax is more dependent on the holding time, given that its permeability is lower than that of twill reinforcements.

While this research provides valuable insights, the authors acknowledge that manufacturing curved thermoplastic composites, commonly required in practical applications, would necessitate distinct production methods. This would likely involve utilizing two-sided molds rather than the single-sided molds used in their experiments, and potentially incorporating autoclaves into the process [48]. Furthermore, the authors overlook the time-dependent variability of permeability, which is influenced by the natural reinforcement of fibers as they absorb liquid and swell.

The permeability of a porous medium is primarily determined by the characteristics of the medium itself, particularly the size and connectivity of the pores. Consequently, it is typically regarded as an intrinsic property of the porous medium, independent of the fluid phase. Indeed, studies on synthetic fabrics have demonstrated that fluid viscosity has a negligible effect on permeability [49,50,51]. However, as resin infiltrates the natural fibers, it is absorbed, causing fiber swelling and altering the reinforcement’s microarchitecture. This can result in a reduction in pore size, which in turn affects resin flow and thus permeability [52,53]. Given that liquid sorption and swelling vary with the specific liquid in question, it follows that permeability, which has been assumed to be constant for synthetic reinforcements, becomes dependent on the liquid in natural fabrics.

Two primary methods exist for measuring permeability [54,55]: the unsaturated method, which assesses flow front advancement under constant inlet pressure, and the saturated method, which gauges flow rate and pressure difference in a fully impregnated preform. While the distinction between unsaturated and saturated permeabilities in synthetic fibers (e.g., glass, carbon) is well documented [55,56,57,58], with unsaturated values typically lower [54,55], the underlying mechanism differs for natural fibers.

In synthetics, the formation of voids and the partially saturated zone situated behind the flow front exert an influence on the permeability of the unsaturated material. This is influenced by the liquid’s wetting properties and viscosity, which impact the formation of microvoids [56,57,58]. For instance, water’s low viscosity and favorable wettability with hydrophilic flax result in rapid tow and swelling, which further complicate the characterization of permeability behavior, saturation, and minimal voiding. In contrast, oil’s high viscosity and unfavorable wettability lead to slower saturation and a larger partially saturated zone. It is of particular importance to note that, in contrast to synthetic materials, natural fibers display liquid sorption properties.

Nguyen et al. [59] investigated the impact of liquid properties on the permeability of natural fabrics in the context of liquid composite molding. The permeability of the flax woven fabric was compared when saturated with distilled water and engine oil, respectively, using the fabric as a model system. The liquids were selected on the basis of their contrasting properties: water exhibits low viscosity and high wettability with flax fibers, while engine oil has high viscosity and poor wettability. It is of particular importance to note that the study revealed the significant impact of fiber swelling on permeability. The authors discovered that, in contrast to synthetic fabrics, the saturated permeability of flax fabric was markedly influenced by the type of impregnating liquid. Specifically, the permeability was found to be higher when engine oil was used in comparison to water. This behavior was attributed to differences in fiber swelling induced by the two liquids. Water exhibits a high sorption capacity with flax fibers, whereas engine oil demonstrates a low sorption affinity. The authors proposed a modified Kozeny–Carman model to accurately predict the saturated permeability of natural fabrics, incorporating the effects of fiber swelling through a dynamic effective fiber volume fraction. The model is capable of predicting the saturated permeability of natural fabrics irrespective of the impregnating liquid. The results underscore the necessity of characterizing resin permeability in relation to the type of reinforcement with which it interacts during the impregnation stage of the consolidation process.

It is also important to consider that natural fibers may not be fully wetted during standard manufacturing processes due to their tightly packed fibril structure. The implementation of enhanced fiber pre-impregnation methodologies can facilitate improved fiber wetting, thereby reinforcing the mechanical interlocking between fiber and matrix [60]. 

### 2.3. Chemical Pre-Treatment of Fibers

**Figure 3:**
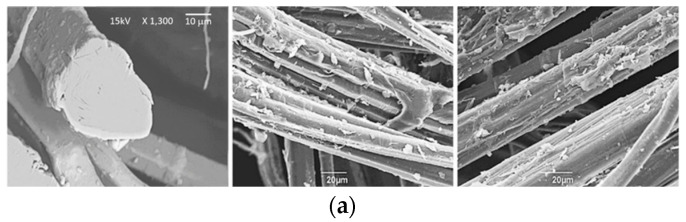

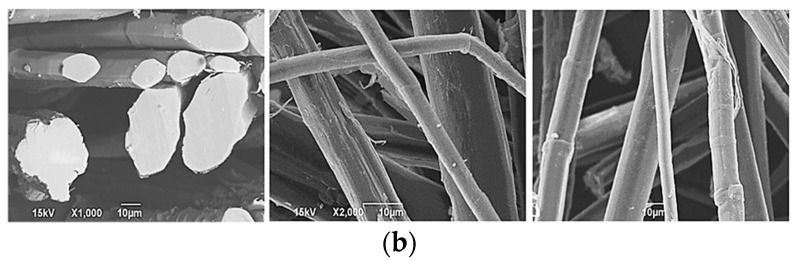
SEM images of flax fibers: (**a**) untreated fibers (**b**); fibers after alkali treatment [35].

Natural fibers are inherently hydrophilic, exhibiting high moisture absorption and poor interfacial bonding with hydrophobic matrices [61]. The incorporation of a third material with intermediate properties is often an effective means of resolving compatibility issues. Chemical treatments are indispensable for modifying the hydrophilic nature of these fibers and enhancing their compatibility [62]. The selection of an appropriate chemical treatment is dependent upon a number of factors, including environmental considerations and cost implications. Although natural fibers possess advantageous characteristics such as high strength, stiffness, and structural stability, their dimensional instability and moisture absorption can impede their adhesion to matrix materials [61]. As previously mentioned, lignocellulose natural fibers are composed of cellulose, hemicelluloses, lignin, pectin, and waxy elements, with cellulose being the primary component that contributes to their strength and structural properties [63]. The bonding between cellulose–hemicellulose chains and lignin–pectin provides the fiber’s structural integrity [64]. However, the chemical disparity between natural fibers and matrix materials can lead to ineffective load transfer and poor interfacial adhesion [65]. A variety of chemical treatments, including alkali, acetylation, silane, maleated coupling agents, acrylation, permanganate, benzoylation, isocyanate, peroxide, sodium chlorite, and stearic acid, have been demonstrated to enhance interfacial bonding by modifying the fiber surface (see Figure 3) and promoting improved compatibility with the matrix [66]. These treatments can enhance the strength properties of composites by eliminating weak boundary layers, creating thin and flexible interfacial regions, forming cross-linked interphase compression regions, improving wettability, developing covalent bonds, and altering the substrate surface.

Mattlet A. et al. [67] investigated the impact of processing and matrix parameters on unidirectional flax–PP composites fabricated via thermocompression. In this study, the researchers discovered that the incorporation of maleic anhydride-grafted polypropylene (MAPP) as a coupling agent markedly enhances the interfacial adhesion between flax fibers and the polypropylene matrix. The study revealed that while a 3 wt% addition of MAPP (maleic anhydride-grafted polypropylene) leads to notable improvements in elastic modulus and tensile strength, a critical concentration of at least 5 wt% is required. Beyond this threshold, additional MAPP additions do not result in significant improvements in mechanical properties, indicating potential limitations due to the self-entanglement of polypropylene chains within the compatibilizer. Although the study provides a comprehensive analysis of MAPP’s impact on the composite’s mechanical properties and morphology, it fails to address the potential long-term effects of MAPP, including its durability, aging behavior, and environmental impact. Furthermore, the study does not examine the thermal stability of the material or define the degradation mechanisms.

### 2.4. Thermal Pre-Treatment of Fibers

Unlike synthetic fibers, natural fibers require thermal treatment in a climatic chamber to eliminate moisture content. As previously stated, the moisture sensitivity of natural fibers necessitates careful control prior to processing to prevent composite defects such as porosity and poor fiber–matrix adhesion.

Prasad and Sain [68] subjected hemp fibers to thermal treatment in an inert atmosphere at 180 °C, which surpassed the glass transition temperature of lignin. This resulted in the migration of lignin to the fiber surface, enhanced hydrophobicity, and fiber opening in both the longitudinal and transverse directions. Such treatment under inert conditions resulted in enhanced tensile strength and stiffness, potentially due to an increase in fiber count and a reduction in diameter caused by fiber opening. In contrast, thermal treatment in air resulted in a reduction in strength due to the oxidation of the fibers.

Moudood A. et al. [69] investigated the impact of moisture content in flax fiber fabrics on the quality of epoxy-reinforced composites. The study revealed that dimensional stability was adversely affected in composites comprising flax fabrics subjected to high relative humidity (RH). Warping was observed as a consequence of the swelling and subsequent shrinkage of the fibers during the absorption and desorption of moisture. A moisture content threshold between 7% and 14% was identified where residual stresses from shrinkage resulted in significant deformation.

The formation of voids in the composite materials was clearly evident at the microstructural level. While dry fabrics yielded composites with minimal void formation, higher RH levels resulted in increased porosity, particularly in the surface region. The observed porosity was attributed to the formation of water vapor during the curing process, and matrix cracking was observed in composites made with the most humid fabrics. It is important to note, however, that this study did not consider the effects of repeated moisture absorption and desorption cycles (hygro-thermal cycling). Such cycles can induce internal stresses and micro-damage, ultimately impacting the composite’s long-term performance. A more realistic understanding of the behavior of composites with different moisture contents in service can be gained by assessing the influence of hygro-thermal cycling.

A distinct thermal treatment for natural fibers is steam explosion, a process that employs high-temperature steam to separate fiber bundles into smaller, cleaner fibers with rough surfaces [70]. This technique can be combined with chemical agents to simultaneously treat natural fibers chemically and mechanically, as demonstrated with flax [71].

## 3. Polypropylene

The versatility of PP extends to its processing methods, encompassing extrusion, thermoforming, and injection molding [72]. The widespread use of PP as the preeminent polyolefin material can be attributed to its remarkable attributes, including low density, high melting point, favorable mechanical properties, relatively high-temperature resistance, excellent processability, and commendable impact resistance [73,74,75,76,77]. In addition, PP’s morphological structure can be tailored through the use of fillers, reinforcing agents, and polymer blending, resulting in materials with enhanced characteristics [78,79]. Its recyclability and exceptional chemical stability [80,81] make it a cost-effective choice for applications demanding long-term durability. Glass fiber-reinforced PP composites, for example, exhibit superior resistance to water, salt solutions, and freeze–thaw cycles, making them ideal for structural applications [82]. In the medical field, PP’s combination of versatility, processability, and compatibility with sterilization methods has led to its increased adoption, providing a cost-effective alternative to other materials [83].

A significant drawback of polypropylene is its susceptibility to degradation at temperatures below 0 °C, limiting its applicability in cold environments [81]. While it exhibits favorable properties at ambient temperatures, its limitations necessitate careful consideration for applications requiring low-temperature performance.

The production of PP involves a multi-step process, with the majority of propylene monomer globally sourced from the steam-cracking process using naphtha, a valuable fraction of crude oil [84]. Propylene, often a byproduct of ethylene monomer production, is collected through dedicated propylene plants integrated into naphtha cracking facilities. Alternative production methods include gasoline refining and the dehydrogenation of propane [85].

There are three primary types of PP: homopolymer PP (HPP), random copolymer (RCP), and impact copolymer (ICP) [73]. HPP consists solely of propylene monomers in a semi-crystalline structure [81]. RCP incorporates ethylene as a comonomer, altering the polymer’s properties [86]. ICP is a blend of HPP and RCP, providing enhanced impact resistance [87].

PP also exists in three stereoisomeric configurations: isotactic, syndiotactic, and atactic, based on the arrangement of methyl groups along the polymer backbone [88]. Isotactic PP has a higher melting point (171 °C) compared to syndiotactic PP (130 °C) [80].

HPP is the most widely used PP type [73]. Its structure comprises crystalline and non-crystalline regions. The non-crystalline phase contains both isotactic and atactic PP, with the isotactic component capable of slow crystallization over time [81]. In RCP, ethylene disrupts the regular PP structure, leading to reduced crystalline uniformity. A higher ethylene content correlates with decreased crystalline thickness and lower melting point [80]. ICP combines HPP and RCP, offering improved impact resistance at low temperatures. The RCP component, often referred to as the rubber phase, has a higher ethylene content (45 ÷ 65%) than the overall ICP [73].

Copolymer polypropylene exhibits superior impact resistance, stiffness, and processability compared to homopolymer polypropylene. Both materials, however, demonstrate limited low-temperature impact strength. 

While polypropylene offers numerous benefits, its applications can be constrained by several factors. These include susceptibility to UV degradation, poor low-temperature impact resistance, challenges in joining, vulnerability to chlorinated solvents and aromatics, accelerated oxidative degradation in the presence of certain metals, and flammability, which can be mitigated through the use of flame retardants.

With respect to the thermoset resin, thermoplastic processing is significantly faster than thermoset processing [89]. Additionally, thermoplastics can be readily joined using various welding techniques, including resistance welding, vibration welding, and ultrasonic welding. Thermoplastics, including PP, offer several advantages over thermoset polymers in biocomposite fabrication. These advantages encompass

- Low processing temperatures;
- Design flexibility;
- Ease of molding complex parts.

However, PP, primarily derived from petrochemicals, faces sustainability concerns [80,90]. Although mechanical recycling is preferred over composting for plastic waste, repeated recycling can compromise the mechanical properties of PP composites. Interestingly, natural fiber reinforcements in PP composites exhibit superior retention of mechanical properties throughout recycling cycles compared to glass fiber reinforcements [90]. This advantage stems from the inherent flexibility of natural fibers and their ability to withstand external mechanical forces, making them a more sustainable option.

Furthermore, despite its strengths, PP’s inherent flammability necessitates incorporating flame-retardant additives to mitigate fire risks [91]. The melting point of PP, determined using differential scanning calorimetry, typically falls within a range due to variations in crystallinity [92]. While perfectly isotactic PP exhibits a melting point of 171 °C, commercial isotactic PP, with crystallinity ranging from 40% to 60%, melts between 160 °C and 166 °C [93]. Syndiotactic PP, possessing a crystallinity of 30%, has a melting point of 130 °C. While challenges related to flammability and sustainability persist, ongoing research and development efforts continue to optimize PP’s performance and explore avenues for enhancing its sustainability profile.

**Figure 4:**
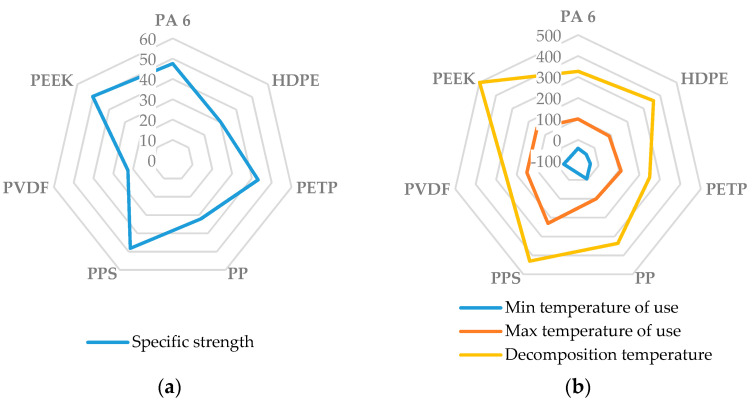
Illustration of various semi-crystalline polymers: (**a**) specific strength, expressed as N·m/g; (**b**) minimum, maximum, and decomposition temperatures in °C.

Thermoplastic polymers, including polypropylene, polyamides (PAs), polyethylene (PE), and polycarbonate (PC), are predominant materials in the transportation sector [94]. Among these, PP stands out as a particularly promising candidate for biocomposite applications. Figure 4 presents a radar graph of the specific strength (tensile strength over density) and the minimum, maximum, and decomposition temperatures of various thermoplastic polymers employed in bio-composites. 

The dominance of PP in the realm of biocomposites is further underscored by its exceptional suitability as a matrix material for composite fabrication. Its compatibility with natural fibers is particularly noteworthy, making the reinforcement of PP with fibrous polymers a promising avenue for creating natural–synthetic polymer composites [80,95,96,97]. 

The application of coupling agents plays a pivotal role in enhancing the performance of natural fiber-reinforced polypropylene (PP) composites, broadening their potential applications. Maleic anhydride grafted polymers (MA-g-PP or MA-g-PE) are particularly effective at improving interfacial adhesion between natural fibers and polymer matrices. Research has shown that even small quantities of these functionalized polymers can significantly enhance fiber–matrix bonding, resulting in increased composite strength [98,99]. For instance, the incorporation of MA-g-PP in nano-kenaf–PP composites boosts tensile strength, elongation, and impact resistance by strengthening interfacial adhesion [100]. Similarly, in cellulose-based composites, low graft concentrations of MA-g-PP (0.25–0.5 wt%) can achieve maximum tensile strength, though excessive free MA monomers may interfere with fiber–matrix interactions [101]. In flax–MAPP blends, the use of MAPP improves the fiber–matrix interface and melt flow properties, resulting in a 15% increase in strength and 25% increase in modulus compared to composites made with standard PP [102]. These findings highlight the effectiveness of MA-grafted polymers in enhancing the mechanical properties of natural fiber-reinforced composites.

Silane agents, such as aminopropyltrimethoxysilane and aminopropyltriethoxysilane, have also been shown to improve interfacial adhesion and mechanical properties, although MAPP remains more effective in comparison [103]. Despite the availability of coupling agents that enhance adhesion between polypropylene and natural fibers, the primary factors governing optimal adhesion are rooted in the processing parameters. Therefore, this review paper centers on these critical processing conditions and their fundamental role in optimizing fiber–matrix adhesion in natural fiber-reinforced composites.

Polylactic acid (PLA) emerges as a promising alternative to polypropylene due to its biodegradable nature and potential to enhance fiber–matrix adhesion in natural fiber composites [104,105]. While PLA composites exhibit superior mechanical properties and load transfer [78], PP composites offer lower viscosity and better fiber wetting, crucial for processing efficiency [106]. Additionally, PLA’s brittleness compared to PP’s ductility [106] may pose limitations in applications requiring impact strength.

As mentioned, from an environmental perspective, PLA’s biodegradability and renewable resource origin provide a more sustainable option compared to PP [107]. However, PLA’s limitations in heat resistance and susceptibility to degradation in harsh conditions [104,108] hinder its suitability for long-term, durable applications. Addressing these challenges through further research and development is essential for the successful transition from PP to PLA composites.

While PLA has gained significant attention, biodegradable polymers such as polyhydroxybutyrate (PHB), polyhydroxyalkanoates (PHA), polycaprolactone (PCL), polybutylene adipate terephthalate (PBAT), thermoplastic starch (TPS), and polycaprolactone (PCL) have also emerged as viable alternatives to polypropylene. 

PLA and PHA offer a trade-off between high strength and low elongation, whereas TPS, PCL, and PBAT prioritize ductility over ultimate tensile strength [109]. PBAT’s economic viability is constrained by its higher production costs. PBAT/TPS blends, when combined with reactive compatibilizers, offer a potential solution to balance cost-effectiveness with desirable material properties [110].

PHB, a bacterial polyester, demonstrates biodegradability equivalent to that of traditional plastics while maintaining comparable mechanical properties. 

While natural materials are often perceived as environmentally friendly, they can pose significant environmental risks, as demonstrated by Laranjeiro F. et al. [111]. The study found that PHBv leachates were highly toxic to all tested marine species, with microalgae being the most sensitive. This toxicity was linked to the presence of harmful compounds such as 2,4,6-trichlorophenol and estragole, as well as the release of micro- and nanoparticles and chemical additives. PLA leachates showed lower toxicity compared to PHBv but still caused slight toxicity in microalgae and mussels, with the detection of bisphenol A diglycidyl ether, a possible endocrine disruptor. Past research on PLA toxicity has been inconsistent. PP leachates exhibited minimal to no toxicity across species, and its toxicity levels were comparable to PLA based on SSD curves. 

## 4. Processing Parameters and Hot-Press Method

### 4.1. Temperature and Holding Time

The thermal sensitivity of flax fibers, as evidenced by their susceptibility to degradation at elevated processing temperatures [112], represents a significant limitation to their potential for high-performance composite applications. This degradation compromises the mechanical properties of the fibers, ultimately affecting the overall performance of the fabricated composites [1,113] 

In addition to temperature, the duration of exposure to the maximum formability temperature has also been identified as a critical factor influencing the integrity of vegetal fibers and processing quality. While some researchers recommend brief, high-temperature processing, others suggest longer processing times at lower temperatures. This has resulted in conflicting recommendations in the literature [1,113,114,115]. Conversely, extended holding times at elevated temperatures offer certain advantages. It is noteworthy that prolonged exposure to elevated temperatures facilitates the reduction of porosity. This occurs as a result of the extended exposure, which provides sufficient time for the coalescence and expulsion of air voids that are introduced into the matrix during the processing stage [116]

K. Van et al. [117] analyzed the influence of material and process parameters on the mechanical properties of both unidirectional (UD) and multidirectional (MD) needle-punch nonwoven composites, exploring the influence of flax treatments, matrix modifications, and processing parameters on the resulting composite materials. The influence of processing parameters, including pressing temperature and time, has been investigated. Three temperature levels were employed and the effects of temperature (190, 200, and 210 °C) and time (two levels) were examined. The objective was to optimize composite properties by varying the processing time (3.5 and 5 min). The application of a double needling process results in the production of thinner panels when the pressing temperature is set to 190 °C. This trend reverses at higher temperatures, with the exception of a five-minute press at 200 °C. It was postulated that the enhanced mechanical cohesion of twice-needled nonwovens is responsible for this behavior. At lower temperatures, inadequate material penetration hinders the pressing process, allowing the nonwoven’s inherent elasticity to influence the final panel thickness. Consequently, nonwovens that have undergone a double needling process, due to their greater cohesion, exhibit the thinnest panels. While pressing temperatures of 200 °C were sufficient for achieving the desired properties, higher temperatures did not result in significant improvements and may potentially induce degradation of the flax fibers. However, the precise temperature threshold for degradation and its impact on composite properties remain unclear. It is important to note that the study has a notable limitation in that it explores the effects of high-temperature processing on flax fiber degradation to a relatively limited extent. Although the potential for degradation at elevated temperatures was acknowledged, the specific temperature ranges at which this becomes a critical factor remain undefined. It is of particular importance to note that the manufacturing of natural fiber-reinforced polymers (NFRPs) is highly susceptible to processing temperature, due to the decomposition of cellulose at 200 °C [36]. While substantial weight reduction occurs above 220 °C (110 °C), color alterations are already discernible at 180 °C [118]. Indeed, as demonstrated by Velde and Baetans [119], temperatures exceeding 180 °C should generally be avoided during composite manufacturing, particularly when the process is prolonged. This study is in accordance with the research conducted by Gourier et al. [120]. The study of the impact of thermal cycles on flax fibers revealed that significant alterations to the fiber structure and intercomponent interactions occur at 250 °C.

In a related study, Van de Velde and Kiekens [117] investigated the influence of time, temperature, and MAPP treatment on flax–PP laminates produced via compression molding. Additionally, the study encompassed an array of flax fabrics derived from fibers subjected to diverse processing techniques, including retting, boiling, bleaching, and needle-punching. The optimal processing temperature for fibers was determined to be 190 ÷ 200 °C, with boiled flax treated with MAPP yielding the best mechanical properties.

### 4.2. Compaction Pressure

As previously mentioned, while thermoplastic polymers require elevated temperatures for melting, natural fibers, such as flax, are susceptible to degradation under such conditions. This results in a complex interplay between processability, particularly the permeability of the resin through the reinforcement, and material integrity. The issue can be resolved by maintaining a temperature below a critical threshold to prevent substantial degradation of the reinforcement and by regulating the compaction pressure.

Consolidation pressure exerts a significant influence on composite morphology, as it effectively reduces porosity and enhances mechanical properties. Rama-Krishnan et al. [121] demonstrated this by manufacturing commingled flax–PP composites using fast induction-heated compression molding. An increase in pressure from 20 to 40 bars resulted in a notable reduction in porosity, from 3.5% to 1.1%. This reduction is attributed to the compression or penetration of fiber lumens by the polymer resin at elevated pressures. Furthermore, elevated pressures not only result in the collapse of the lumen within elementary fibers but also facilitate the compaction of the laminate, thereby reducing the number of voids. Microscopic analysis conducted by Mattlet et al. [67] revealed that higher pressures correlate with decreased fiber dispersion, characterized by fewer but larger fiber bundles. This phenomenon is likely due to reduced fiber permeability or increased compaction of fiber bundles at higher pressures [19,122].

While pressure is typically regarded as a pivotal factor in reducing void content in composite manufacturing, some studies have indicated that its efficacy may reach a plateau at specific pressure thresholds. Klinkmüller et al. [44] demonstrated that alterations in compaction levels and holding durations have a negligible effect on the consolidation process of GF/PP commingled yarn-based composites manufactured through compression molding when operating at elevated processing temperatures. At pressures above 2 MPa and temperatures approaching the polymer melting point, the impregnation of the fibers is largely unaffected, indicating that the primary influence of the compaction pressure is on the fiber volume fraction rather than on the quality of consolidation. Significant discrepancies in void content were predominantly discerned between atmospheric and low-pressure (0.375 MPa) processing conditions. The temperature exhibited the most pronounced effect on void reduction, with the greatest reduction occurring at temperatures up to 175 °C, and diminishing returns at higher temperatures.

### 4.3. Cooling Rate

One of the primary challenges associated with natural fiber-reinforced thermoplastic composites is the weak interfacial bonding between the fiber and the matrix. The quality of the interfacial adhesion significantly affects the overall performance of the composite. This issue primarily arises from the intrinsic incompatibility between hydrophilic fiber surfaces and hygroscopic polymeric matrices. This incompatibility impedes effective fiber–-matrix bonding, resulting in heterogeneous material systems with compromised properties [123,124]. As a result, the debonding of fibers creates defects that reduce the composite’s effective cross-sectional area, leading to a decline in mechanical strength. Moreover, inter-fiber hydrogen bonding can facilitate the agglomeration of fibers, impeding uniform fiber dispersion and negatively affecting both composite strength and aesthetics [123,125].

To enhance composite properties and overcome limitations associated with poor fiber–matrix adhesion, interfacial modification techniques are crucial. As previously stated, the most commonly utilized techniques to enhance interfacial shear strength and, subsequently, to improve overall mechanical performance include the application of compatibilizers to either the fiber surface (e.g., silanes, stearic acid) or the matrix (e.g., maleic anhydride), as well as other methods such as pre-impregnation, chemical reactions, and plasma treatments [60,123,126,127].

In addition to the incompatibility of resin reinforcement, the cooling rate has also been demonstrated to exert a significant influence on the adhesion between fibers and the matrix. Indeed, cellulose is prone to shrinkage as temperatures decrease [128], and semi-crystalline polymers such as polypropylene, nylon, and polyethylene experience appreciable shrinkage upon transition from the liquid to solid phase [19]. This thermal contraction, in conjunction with the lack of robust interfacial adhesion between the fiber and the matrix, may result in the formation of voids at the interface. Furthermore, the thermal degradation of flax fiber can exacerbate this issue by increasing the void content in the aforementioned region. Conversely, a slower cooling rate results in prolonged exposure to elevated temperatures, which has a detrimental impact on the microstructure and mechanical properties of the composite [121,129]. A slower rate of temperature decrease facilitates the formation of volatile byproducts resulting from water evaporation and the degradation of fiber components such as pectin [112,119]. It is therefore essential to conduct a detailed investigation into the optimal cooling rate in order to ensure the preservation of the component’s mechanical integrity.

While cooling rate is often linked to unfavorable outcomes, meticulous regulation of this variable can enhance the interfacial bonding between fibers and matrices. Zafeiropoulos N. et al. [130] investigated the impact of cooling rates on the interfacial strength of flax fiber/polypropylene composites. Two distinct varieties of flax fibers were utilized in the study: dew-retted (DR) and green flax (GR). The study’s primary conclusion is that slower cooling rates result in enhanced interfacial strength in both types of flax fiber composites. This was demonstrated by the shorter fragment lengths observed in the fragmentation tests for samples cooled at slower rates. The study acknowledges the existing controversy in the literature regarding the effect of cooling rates on interfacial strength. The findings of studies conducted by Moon [131] and Youssef and Denault [132] have reported that fast cooling increases interfacial strength in glass flax–PP composites, attributing this to either increased contraction forces or a higher proportion of the amorphous phase in the matrix. However, Ye et al. [133], Nielsen and Pyrz [134], and Gao and Kim [135] have found that slower cooling leads to higher interfacial strength in carbon fiber composites with various matrices. This discrepancy highlights the complexity of the interfacial phenomena and the varying influences of cooling rates depending on the specific fiber and matrix materials involved. The authors propose two potential explanations: enhanced adsorption of crystalline PP on the fiber surface during slower cooling and reduced residual stresses at the interface. However, they acknowledge the lack of data on specific flax fiber properties as a limitation in providing a definitive explanation for the observed phenomenon. Moreover, the study demonstrates the formation of a transcrystalline layer in samples subjected to slower cooling rates. The presence and thickness of this layer exhibit variability between DR and GR fibers, thereby introducing complexity in the analysis of the sole effect of cooling rates. Prior research indicates that transcrystallinity can exert a considerable influence on interfacial strength [136]. Furthermore, the relationship between transcrystalline layer thickness and interfacial strength appears to differ between the two fiber types, with a notable impact observed in GR flax but not in DR flax. Although this study demonstrates the beneficial effects of slower cooling rates on flax–PP composites, the underlying mechanisms remain unclear. Further research is required to elucidate the specific interactions between the flax fibers and the polypropylene matrix under different cooling conditions.

### 4.4. Hot Press Method

Hot pressing is a suitable method for the production of composites, necessitating only two heated platens to simultaneously compress and heat the fiber and matrix. However, regulating the viscosity of the matrix during the pressing and heating processes represents a significant challenge, particularly when working with thicker samples [137]. The optimal viscosity of the matrix is of great importance for the correct impregnation of the fibers while simultaneously preventing the expulsion of the material [138]. Due to their filamentous nature, natural fibers require additional time for wetting [139]. Accordingly, precise control of viscosity, pressure, holding time, and temperature is essential for achieving high-quality composites, taking into account the specific fiber type, matrix properties, and dimensions of the sample in question [140].

Defects induced by the manufacturing process, such as residual stresses, voids, warping, fiber breakage, sink marks, and scorching, can have a significant detrimental impact on the mechanical properties of the composite material [141]. It is essential to optimize the process, material, and geometric parameters in order to minimize the formation of defects. In the case of biodegradable polymers, it is imperative that the processing temperatures remain below 200 °C in order to prevent degradation [142,143]. It is of the utmost importance to ensure effective heat transfer from the sample surface to the core in order to prevent overheating or underheating. This necessitates a careful analysis of the temperature gradient.

The non-uniform temperature distribution that occurs within molded parts has the potential to induce residual stresses, particularly in the thicker sections. The rate and profile of cooling exert a considerable influence on the relaxation of stresses [144]. High pressures are essential for components with deep features, such as ribs and bosses [145]. However, the production of thick parts is challenging due to heat conduction limitations that characterize flax–PP materials

Dobah et al. [1] investigated the factors influencing the thermoformability of flax–PP commingled composites through the hot press forming method. The objective of the study was to examine the influence of temperature, dwell time, and pressure on the mechanical and physical properties of the material. The authors posit that the thermoforming process is controlled by five key variables: maximum temperature and duration, pressure, fabric weave, and laminate size. These factors were examined individually to ascertain their respective effect. Higher temperatures and longer processing times resulted in improved material flow and consolidation, which enhanced the load-bearing capacity and reduced fiber imperfections. However, excessive heat resulted in material degradation, as evidenced by the darkening of the laminates, which consequently compromised the mechanical properties. Conversely, at a temperature of 175 °C, extended dwelling times and elevated pressures result in fiber dislocation during outward matrix flow, attributed to its high viscosity. Composites produced at temperatures above 185 °C exhibited superior mechanical properties but displayed reduced flexibility in comparison to those manufactured at higher temperatures (190 and 200 °C). An increase in pressure resulted in enhanced tensile and flexural performance, which was attributed to improved fiber stretch and compactness. However, the impact of pressure variations on failure modes was not statistically significant. The authors acknowledge the potential of flax–PP composites but also recognize the limitations of their research methodology. These include the equipment’s inherent limitations, such as the absence of a cooling stage and imprecise temperature control. Additionally, the study examined only a limited range of parameter combinations. To gain a comprehensive understanding of the potential of flax–PP composites, it is essential to explore a wider array of parameters.

Similar results were achieved by Zhang J. et al. [115], who focused on determining the optimal blending ratio of flax and PP fibers, molding temperature, and molding time for a hot press processing in order to maximize tensile and bending strengths. The optimal processing parameters were identified as a 50:50 flax–PP fiber blending ratio, a molding temperature of 181 °C, and a molding time of 50 min. An increase in flax fiber content initially resulted in enhanced mechanical strength. However, excessive amounts impeded PP resin flow, leading to stress distribution inconsistencies and a subsequent reduction in strength. The molding temperature had a significant impact on the composite strength. Insufficient temperatures prevented the proper melting of the PP fibers, while excessive temperatures degraded the PP matrix. The molding time had a significant impact on the interaction between the flax fibers and the PP matrix. Insufficient time resulted in poor bonding, while excessive time led to reduced mechanical properties, matrix cracking, and fiber degradation.

As previously stated, the processability of flax–PP composites using thermocompression involves several key parameters that influence the final material properties. Mattlet et al. [67] conducted a comprehensive investigation into the influence of six pivotal parameters—temperature, thermocompression duration, MAPP content, pressure, cooling speed, and exit temperature—on the morphological and mechanical characteristics of unidirectional flax–PP composites processed via thermocompression press forming. The researchers discovered that elevated temperatures and extended processing times resulted in a reduction in tensile strength, which they attributed to fiber degradation. Composites processed at 200 °C for 11 min showed a 22% drop in tensile strength compared to those processed for 3 min. Thermogravimetric analysis confirmed fiber degradation at higher temperatures. MAPP, which is known to improve fiber–matrix adhesion in natural fiber composites, enhanced the interfacial adhesion, increasing tensile strength by 13% at 3 wt%. Pressure did not significantly affect the mechanical properties of the material; however, it did lead to a reduction in fiber dispersion. Ultimately, the application of lower cooling rates resulted in an increase in porosity and a reduction in tensile strength. However, exit temperatures did not have a significant impact on the observed properties. While this study provides valuable insights, it is limited in scope in that it neglects to consider fiber architecture, matrix variations, and a wider range of process conditions. Further research is required to gain a full understanding of the interactions between process parameters.

Ramakrishnan et al. [146] conducted a comprehensive study on the effects of processing parameters (temperature, pressure, time) on the mechanical and damping properties of flax–PP commingled composites fabricated via thermocompression molding. The results demonstrated a notable correlation between processing conditions and composite performance. An increase in fiber content resulted in a notable enhancement in both the elastic modulus and ultimate strength. However, elevated temperatures (240 °C) had a detrimental effect on the strength of the composite, resulting in a nearly 35% decrease in comparison to composites processed at 200 °C. Furthermore, the failure strain was observed to be lower at 240 °C (1.3%) than at 190 °C (2.4%). The damping factor was also found to be sensitive to microstructure and processing conditions, with composites manufactured at 240 °C exhibiting reduced damping. These findings indicate that thermal degradation, alterations to the fiber–matrix interface, and fluctuations in fiber volume fraction at elevated temperatures impact the composite’s vibration response. Future research should investigate a broader range of processing parameters, explore additional mechanical properties, analyze moisture absorption, thermal stability, and interface characteristics, and evaluate different flax prepregs and matrix properties to gain a more in-depth understanding of flax-reinforced polypropylene composites.

Table 2 provides a concise overview of the primary benefits and drawbacks of the hot press technique for processing flax–PP composites.

## 5. Conclusions

This review illustrates that flax–polypropylene composites are a promising alternative to traditional synthetic composites, particularly in applications where both mechanical performance and environmental responsibility are of paramount importance. One significant challenge in the processing of flax–PP composites is the poor interfacial bonding between the hydrophilic flax fibers and the hydrophobic polypropylene matrix. This issue can give rise to a number of defects, including the formation of voids, which in turn result in inferior mechanical properties and an increased tendency to absorb water. In conclusion, these deficiencies restrict the composite’s performance and durability. To address this issue, it is necessary to implement optimized manufacturing processes, such as hot press molding.

The primary points highlighted in this review pertain to the use of flax fibers, polypropylene, and processing parameters through hot press consolidation. These are outlined below:

- Flax fibers, which have the potential to serve as a promising alternative to traditional E-glass fibers, offer advantages in terms of density and sustainability. Their comparable mechanical properties and potential for reducing environmental impact make them a viable choice for use in composite materials. The structural complexity, hydrophilicity, and permeability of flax fibers present significant challenges in the creation of composites with hydrophobic matrices, such as polypropylene. These factors require pre-treatment to enhance interfacial adhesion and guarantee appropriate resin impregnation.
- Polypropylene, a material with a wide range of applications in the automotive industry, presents a unique set of characteristics that must be carefully considered during the selection and processing of materials. It is a versatile polymer known for its low density, high melting point, good mechanical properties, excellent processability, and recyclability. However, the petrochemical origin of polypropylene gives rise to concerns regarding its sustainability. Although polypropylene is a popular choice for biocomposites due to its compatibility with natural fibers, its susceptibility to degradation at low temperatures and flammability represent significant limitations that must be addressed.
- Hot pressing has been identified as an efficient and versatile production method for flax–PP composites, offering advantages over conventional methods such as resin transfer molding and compression molding. Nevertheless, even with hot pressing, it is of the utmost importance to optimize process parameters, including temperature, holding time, compaction pressure, and cooling rate. This is essential to achieve the desired material properties with minimal defects. It is therefore essential to exercise precise control over the processing parameters in order to achieve optimal matrix viscosity, prevent material expulsion, and ensure consistent part quality. This entails maintaining an equilibrium between temperature and holding time to facilitate resin impregnation while preventing fiber degradation, meticulously regulating pressure to avert material expulsion, and exercising rigorous control over process variables to minimize defects such as voids, warping, and fiber breakage. However, the use of hot pressing is constrained to the production of components with relatively simple geometries and constant thickness, which limits its applicability to more complex designs.

To fully realize the potential of flax–polypropylene composites, further research is needed to

- Enhance fiber–matrix adhesion using compatibilizers;
- Investigate pre-impregnation techniques for better fiber wetting and resin distribution;
- Optimize hot press molding parameters for specific applications;
- Examine the influence of fiber architecture, matrix modifications, and process conditions on composite properties;
- Improve long-term durability through understanding aging and degradation.

By investigating these research areas, it is possible to develop flax–polypropylene composites with enhanced properties. This development has the potential to facilitate the widespread adoption of these materials as sustainable alternatives to conventional materials, particularly in sectors that require the production of large quantities of eco-friendly components. Nevertheless, the growing necessity for intricate geometries represents a considerable obstacle to this technology. To surmount this limitation, further research and development efforts will be required.

## Figures and Tables

**Table 1:** Side-by-side overview of E-glass and flax fiber characteristics [21].

Property	E-Glass	Flax
Diameter (µm)	8 ÷ 14	10 ÷ 80
Density (g/cm^3^)	2.56	1.40
E-modulus (GPa)	76	50 ÷ 70
Tensile strength (GPa)	1.4 ÷ 2.5	0.5 ÷ 1.5
Elongation to fracture (%)	1.8 ÷ 3.2	2.0 ÷ 3.0
Specific E-modulus (GPa per g/cm^3^)	30	36 ÷ 50
Specific tensile strength (GPa per g/cm^3^)	0.5 ÷ 1.0	0.4 ÷ 1.1

**Table 2:** Benefits and drawbacks of the hot press method for flax–PP composite production.

Hot Press Technology for Processing Flax–Polypropylene Composites	
Advantages	Disadvantages
Relatively simple process requiring minimal equipment. Good for producing parts with a high fiber volume fraction. Offers good control over the thickness and fiber orientation of the final part.	Difficult to control matrix viscosity during processing, which can lead to inconsistent fiber impregnation. Can lead to process-induced defects such as voids, warping, and fiber breakage if processing parameters are not carefully controlled. Limited to manufacturing parts with relatively simple geometries and constant thickness. Heat transfer limitations can make it challenging to produce thick parts.

## Data Availability

No new data were created or analyzed in this study.
